# The Long‐Term Quality of Life Study for Patients Received Chest Wall Reconstruction by Using 3D‐Printed PEEK Implants

**DOI:** 10.1111/1759-7714.70323

**Published:** 2026-06-17

**Authors:** Ge Li, Xiao Liang, Xing Li, Yangfan Huo, Minghai Ma, Yuanquan Zhang, Minglei Zhuo, Lei Wang

**Affiliations:** ^1^ Department of Thoracic Surgery Tangdu Hospital, The Fourth Military Medical University Xi'an China; ^2^ Department I of Thoracic Oncology Peking University Cancer Hospital & Institute Beijing China

**Keywords:** 3D‐printed, chest wall reconstruction, long‐term follow‐up, PEEK, Quality Qf Life

## Abstract

**Background:**

For the repair of chest wall defects, three‐dimensional‐printed PEEK implants show great potential due to their excellent anatomical matching and mechanical properties. However, Quality‐Of‐Life assessments after using these implants in chest wall reconstruction remain underexplored.

**Methods:**

A retrospective cohort study included 20 patients who underwent chest wall defect reconstruction with 3D‐printed PEEK implants at our center between April 2017 and June 2024 and were followed up long‐term. Clinical data collected at various time points included safety indicators (long‐term adverse reactions, blood routine, hepatic and renal function), respiratory function assessments (pulmonary function tests, arterial blood gas analysis), Quality‐Of‐Life indicators (SF‐36 health survey).

**Results:**

Through a median follow‐up of 35.3 months, we found that personalized PEEK thoracic rib implants achieved precise anatomical reconstruction and effective respiratory dynamics maintenance. Complete recovery of preoperative hematological parameters (*p* > 0.05) and liver/kidney function confirmed their biochemical safety. ≥ 1 year after surgery, the restrictive ventilation function of the patients was still lower than that before the operation, and the CO_2_ excretion increased (*p* < 0.05), but the alveolar ventilation function wasn't impaired. Oxygenation was normal (*p* > 0.05). The patient's physical health scores (SF‐36 PCS) remained unchanged from preoperative levels (*p* > 0.05), while psychosocial functioning (SF‐36 MCS) showed significant improvement (*p* < 0.05).

**Conclusions:**

3D‐printed PEEK implants used in chest wall reconstruction exhibited favorable safety and were associated with improved long‐term Quality Of Life. These findings support that PEEK reconstruction may contribute to a combined benefit of disease control, functional preservation, and psychological recovery.

## Graphical abstract

3D‐printed PEEK implants used in chest wall reconstruction demonstrated high safety and enabled patients to achieve better long‐term Quality Of Life.

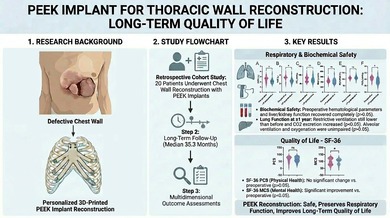

## 1 Introduction

As an essential biomechanical structure, the chest wall maintains thoracic contour and shields intrathoracic visceral organs. Its muscular tissues, together with intrathoracic negative pressure, jointly drive respiratory movement. Chest wall tumors are rare neoplasms predominantly occurring as secondary lesions, while primary chest wall tumors only account for approximately 5% of all cases. Among the primary tumors, malignant ones account for about 60% [1, 2]. Bone and soft tissue sarcomas are the most common primary malignant tumors of the chest wall [3, 4]. If chest wall tumors are not treated in time, they can cause progressive pain, damage to the chest wall structure, and respiratory dysfunction, significantly increasing the risk of malignant tumor metastasis. Thoracic deformity caused by huge defects is prone to lead to body image disorder and social function withdrawal, which impairs the Quality Of Life [5, 6]. With the exception of Ewing sarcoma and solitary plasmacytoma, surgical resection is the first‐line treatment for chest wall tumors. The large chest wall defect caused by the tumor enlargement resection of the chest wall will damage the stability of the chest wall structure and then affect the normal physiological activities of the patient. In severe cases, it will lead to life‐threatening complications such as abnormal breathing [7, 8].

The structure of the chest wall consists of bone tissue, cartilage tissue, muscle, superficial soft tissue, and associated neurovascular components. According to the expert consensus on chest wall mass resection and chest wall reconstruction, when the maximum diameter of the chest wall defect in adults and adolescents exceeds 5 cm, rigid implants are required for chest wall reconstruction [9]. The ideal chest wall reconstruction material should meet the requirements of anatomical restoration and physiological functional restoration. That is, the reconstruction material needs to have a certain degree of rigidity, be able to maintain the normal shape of the chest wall, protect the important organs in the thoracic cavity, and at the same time ensure that the patient's normal respiratory function is not affected [10].

Polyetheretherketone (PEEK), a semi‐crystalline thermoplastic material in the polyaryletherketone family, demonstrates unique performance advantages: Its elastic modulus (3–4 GPa) closely matches human cortical bone (0.76–19.6 GPa), effectively preventing nonunion caused by stress shielding effects. The material's excellent radiation transmittance (CT value≈100 HU, comparable to costal cartilage) ensures postoperative imaging without artifact interference. With thermal stability (glass transition temperature 143°C, melting point 334°C), it meets repeated high‐temperature sterilization requirements. Chemical inertness guarantees long‐term biostability in vivo. At the same time, a large number of in vitro studies have confirmed that PEEK has no obvious cytotoxicity to a variety of cells (such as fibroblasts), and PEEK will not produce toxic and harmful ions and dissolution and other adverse phenomena after long‐term implantation in vivo, and it is stable for a long time, which makes it a good in vivo implant material [11, 12, 13].

Since 2015, our team has been applying additive manufacturing (3D printing) technology to develop personalized titanium alloy thoracic rib prostheses for chest wall defect reconstruction in clinical research [14]. In 2017, we successfully developed a new‐generation 3D‐printed PEEK thoracic rib implant for chest wall reconstruction, aiming to achieve the dual objectives of “anatomical and function restoration”: precise morphological reconstruction and preservation of respiratory dynamics. To date, this technology has been widely adopted in over 20 major tertiary hospitals across China, with over 100 such surgeries completed to date. The safety and effectiveness of PEEK prostheses used in patients with chest wall reconstruction were analyzed in Tangdu hospital. The perioperative safety was reliable, and no malignant adverse reactions were observed. In the long‐term follow‐up, no significant hepatorenal toxicity, hematotoxicity, or respiratory dysfunction related to PEEK implants was detected [15, 16]. However, no long‐term Quality Of Life outcomes have been previously reported in the literature using 3D‐printed PEEK implants for the treatment of patients with chest wall defects. To fill this evidence gap, this study followed 20 patients who underwent chest wall tumor resection and implantation of 3D‐printed PEEK prostheses with a median follow‐up period of 35.3 months to evaluate their long‐term biosafety, respiratory function, long‐term Quality Of Life, etc., to describe the overall long‐term impact of this personalized reconstruction strategy.

## 2 Methods

### 2.1 Research Design

The research team conducted a retrospective clinical analysis of patients who underwent chest wall reconstruction using 3D‐printed PEEK prostheses at the Department of Thoracic Surgery in Tangdu Hospital from April 2017 to June 2024. Clinical data were collected at three defined time points: (1) Preoperative (baseline before the surgery), (2) Perioperative (7–28 days postoperative), and (3) Long‐term postoperative (≥ 1 year postoperative). The study collected baseline clinical data (including basic information, medical history, tumor grading and pathological type, chest wall defect status, imaging results), safety response indicators (including long‐term adverse reactions, blood routine tests, liver and kidney function results), respiratory function assessments (including pulmonary function tests, arterial blood gas analysis), and Quality Of Life (QOL) assessment data (SF‐36 QOL Inventory).

All participants were screened based on unified inclusion criteria as follows: (1) Patients had an expected chest wall defect diameter > 5 cm; (2) Chest wall reconstruction was performed after tumor resection; (3) Postoperative follow‐up duration ≥ 1 year. Exclusion criteria: (1) failure to complete preoperative quality of life assessment. (2) Unable to provide informed consent; (3) No PEEK implantation as chest wall reconstruction plan; (4) Age < 18 years or > 75 years. Additionally, cases with incomplete baseline data and those who voluntarily withdrew were excluded. All participants voluntarily joined the study and signed informed consent forms (Ethics Committee Number: TDL‐201710‐09. Clinical Trial Registration Number: ChiCTR2300078408).

### 2.2 Personalized Prosthesis Manufacturing and Surgical Reconstruction Methods

**FIGURE 1:**
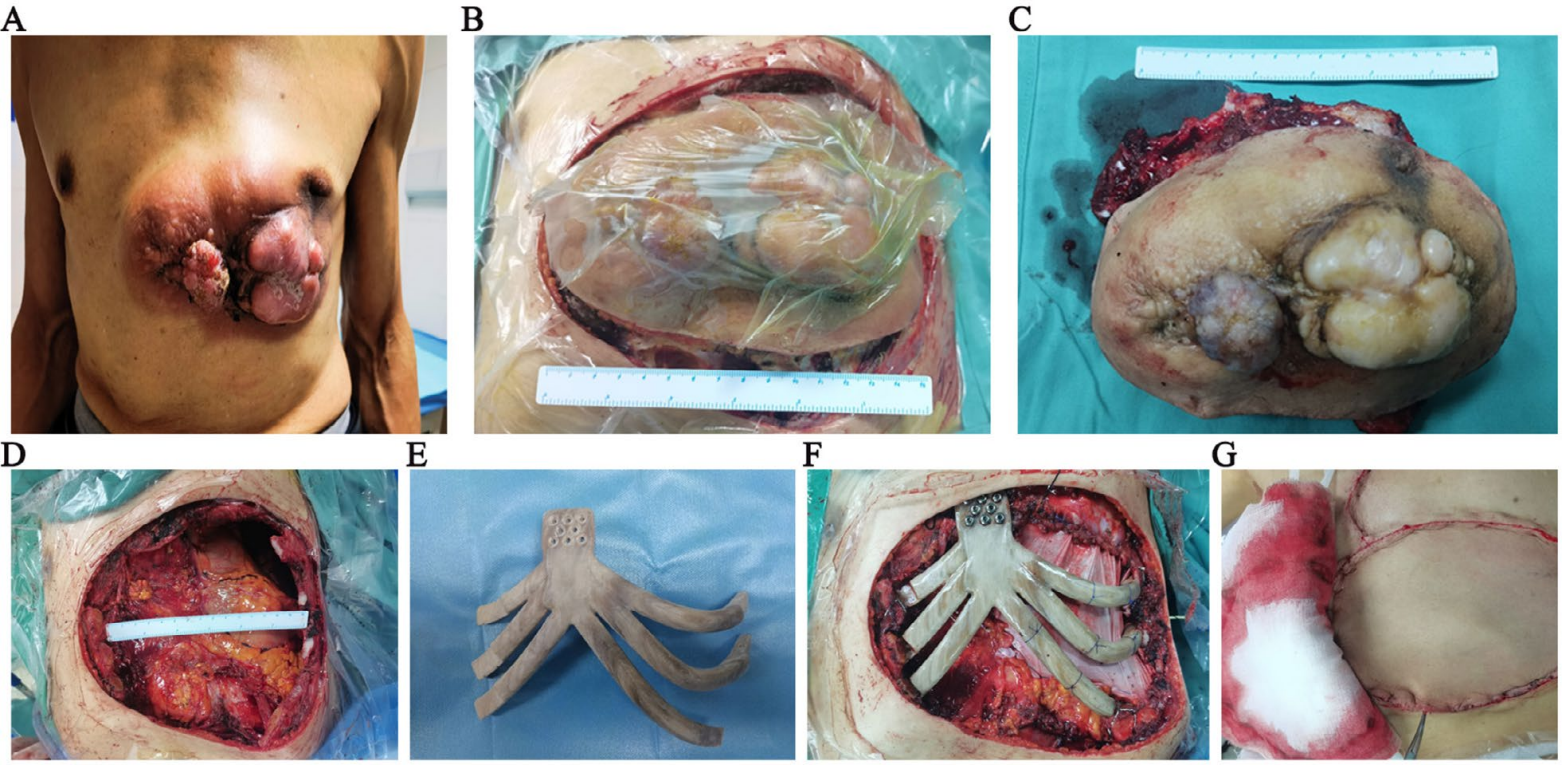
3D‐printed PEEK sternocostal prosthesis used for chest wall reconstruction. (A) Preoperative surface contour of a patient with chest wall tumor. (B–C) Intraoperative tumor resection. (D) Chest wall defect after tumor resection. (E) 3D‐printed PEEK sternum. (F) Implantation of the sternum. (G) Use of free latissimus dorsi musculocutaneous flap to cover the wound surface.

The customized prosthesis was designed through three‐dimensional reconstruction based on preoperative thin‐layer CT scans (layer thickness < 1 mm), with tumor resection margins planned to extend at least 3 cm beyond the lesion [9]. Using natural rib centroid trajectory combined with variable cross‐section scanning (VSS) technology, chest wall prostheses matching the defect were fabricated (Figure 1). Prosthesis types included sternum (full‐length segment/upper segment/inferior segment) and ribs (horizontal/E/vertical type) [13, 15, 17]. Surgical‐grade PEEK material (Victrex, Thornton‐Cleveleys, UK) was selected and manufactured through fused deposition modeling (Jugao‐AM‐Doctor, Shaanxi Juguang, China). Three‐dimensional reconstruction was employed during surgery: (1) Rigid stabilization was achieved through wire ligation (rib–to‐rib connections) and screw fixation (rib‐to‐sternum/sternum remnant); (2) Pericardial patches (Guangzhou Guanhao Biotechnology Co. LTD.) were suspended under the PEEK prosthesis surface, continuously sutured to the residual pleura to reduce pleural cavity dead space; (3) The prosthesis was covered with the muscles and skin of chest wall. Some patients with large soft tissue defects received myocutaneous flap transfer surgery (pectoralis major/latissimus dorsi). Antibiotics were used (Ceftriaxone, 2.0 g, BID, for 3 days) and the bandage was fixed after surgery. At the same time, ultrasound monitoring was performed concurrently until the daily drainage output fell below 10 mL [18, 19].

### 2.3 Safety Analysis

The safety evaluation encompasses three key components: toxicity assessment of the implant, perioperative complications (7–28 days post‐surgery), and long‐term adverse reactions monitoring. Clinical data collection includes blood routine, liver and kidney function results from preoperative, 7–28 days postoperative, and After‐1‐year (≥ 1 year) follow‐up periods, with comparative analysis to assess hematological and renal toxicity. Continuous patient follow‐up is maintained until June 2025, during which telephone or in‐person consultations are conducted to monitor implant‐related complications and adverse reactions.

### 2.4 Respiratory Function Analysis

We collected and compared pulmonary function and arterial blood gas analysis results at the preoperative and postoperative follow‐up period. Pulmonary function tests primarily recorded forced expiratory volume in 1 s (FEV1), forced vital capacity (FVC), the FEV1/FVC ratio, and maximal voluntary ventilation (MVV), while arterial blood gas analysis recorded arterial oxygen partial pressure (PaO_2_) and arterial carbon dioxide partial pressure (PaCO_2_). Preoperative baseline data were collected within 1 week before surgery, and postoperative follow‐up data were obtained at review time points at least 12 months (≥ 1 year) after surgery, with patient follow‐up continuing until June 2025.

### 2.5 Quality of Life (QOL) Assessment

Commonly used quality of life assessment tools include the EORTC QLQ‐C30 [20], FACT‐G [21], EORTC CAT Core [22], PROMIS [23], and PRO‐CTCAE [24]. This study employed the SF‐36 scale, that participants self‐reported their QOL through completing the 36‐item Short Health Survey (SF‐36). The questionnaire evaluates eight dimensions: Physical Functioning (PF), Role‐Physical (RP), Bodily Pain (BP), General Health (GH), Vitality (VT), Social Functioning (SF), Role‐Emotional (RE), and Mental Health (MH). The total score includes Physical Component Summary (PCS) and Mental Component Summary (MCS). PCS is weighted by the scores of PF, RP, BP, and GH, while MCS is weighted by the scores of VT, SF, RE, and MH. The score ranged from 0 to 100, with higher scores indicating better health‐related QOL [25, 26, 27, 28, 29]. There is also Health Transition (HT), which is used for self‐assessment of patients' health status and is not included in the questionnaire score.

### 2.6 Statistical Analysis

The statistical analysis for this study was executed using SPSS version 20, provided by IBM Corp., Armonk, NY, USA and GraphPad Prism 9.0.0. Statistical significance was defined as *p* < 0.05. Continuous variables were reported as mean ± standard deviation, while non‐continuous variables were presented as median/interquartile range.

When conducting data comparisons, the Shapiro–Wilk test is used to examine normality. If *p* ≥ 0.05, the data is considered normally distributed; otherwise, it indicates non‐normal distribution. This study uses paired data from the same patient at different time points for statistical analysis. When comparing two groups of data, for parameters with a normal distribution, paired *t*‐tests are applied to examine changes in measurements across different time points. When dealing with non‐normal distributions, Wilcoxon rank sum tests are used to assess sequential variations. In pairwise comparisons among three groups, repeated measures ANOVA is conducted for normal‐distributed parameters, with Turley's multiple comparisons test serving as post hoc analysis. For non‐normal distributions, Friedman tests are employed, complemented by Dunn's multiple comparisons test for post hoc evaluation.

## 3 Results

### 3.1 Patient Characteristics and Clinical Manifestations

**FIGURE 2:**
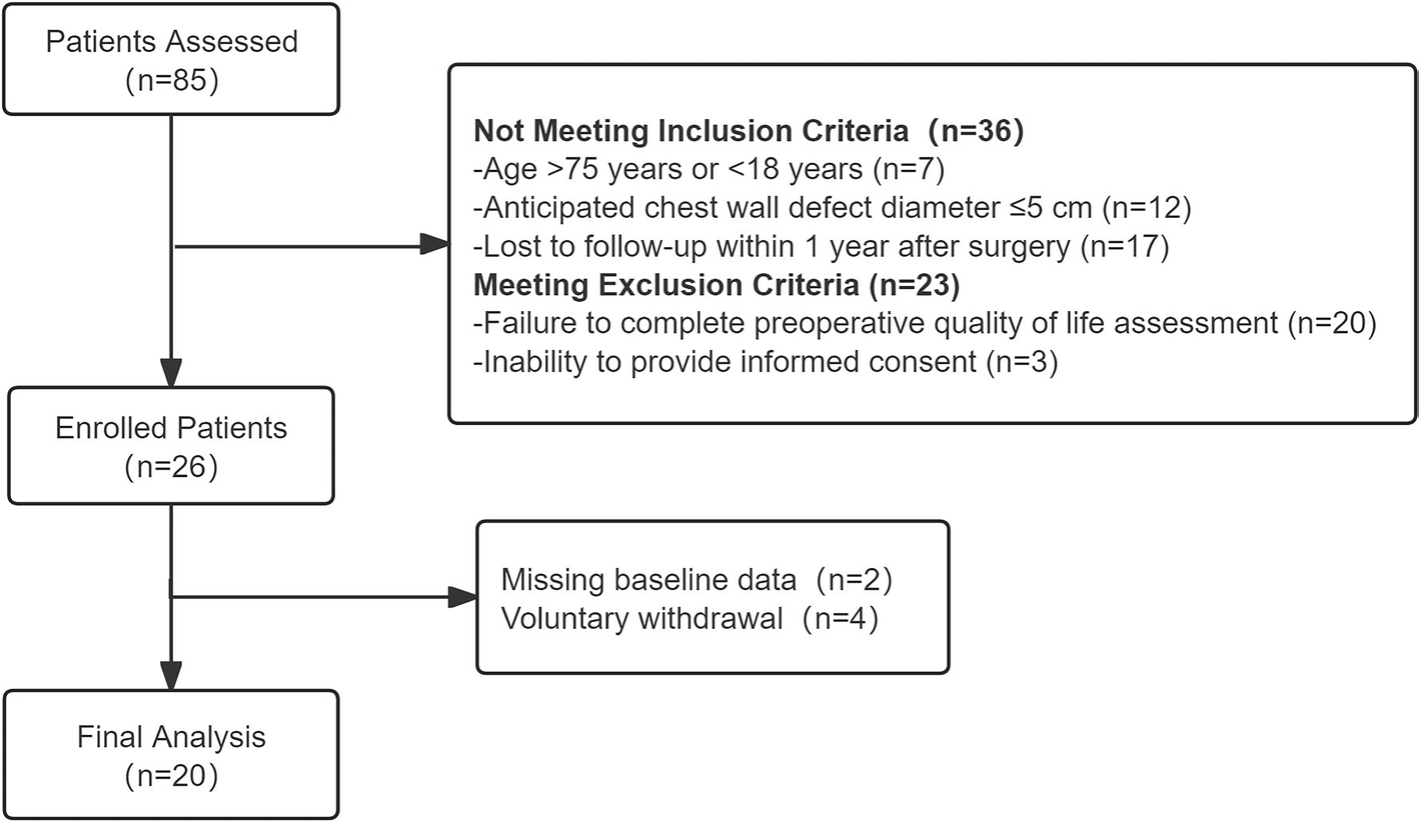
Flow diagram illustrating the process of recruitment.

This study enrolled 20 patients with chest wall tumors who underwent wide excision and 3D‐printed implant reconstruction at our hospital. The process of recruitment is in Figure 2. The patient characteristics, excised chest wall masses, underwent pathological examination, grading, size assessment, detailed pathological classifications, and Ki67 values are listed in Table 1.

**TABLE 1:** Clinical characteristics and surgical data of 20 patients with chest wall masses.

	N	%
Age		
Median/Range	50/30–66	
Sex		
Male/Female	7/13	35.0/65.0
BMI		
Mean/Range	23.8/19.6–27.7	
Location		
Sternum	13	65.0
Ribs	6	30.0
Both	1	5.0
Symptoms		
Asymptomatic	2	10.0
Palpable mess	12	60.0
Pain	13	65.0
Chest tightness	2	10.0
Surgical intervention status		
Initial surgery on the chest	15	75.0
Secondary surgery on the chest	5	25.0
Tumor clinical status		
Primary carcinoma	13	65.0
Metastatic carcinoma	4	20.0
Local recurrence	3	15.0
Tumor dimensions		
Average maximum diameter	6.7 ± 3.2 (cm)	
Average maximum area	38.4 ± 33.3 (cm^2^)	
Chest wall defect dimensions		
Average maximum diameter	18.2 ± 3.2 (cm)	
Average maximum area	254.6 ± 101.0 (cm^2^)	
Tumor grade		
Low proliferation (Ki67 ≤ 10%)	4	20.0
Moderate proliferation (10% < Ki67 ≤ 30%)	4	20.0
High proliferation (Ki67 > 30%)	5	25.0
Unkonwn	7	35.0
Pathology		
Chondrosarcoma (low/high grade)	9	45.0
Adenocarcinoma	2	10.0
Hemangioma	2	10.0
Hemangiosarcoma	1	5.0
Myofibroblastic sarcoma	1	5.0
Serous carcinoma	1	5.0
Plasmacytoma	1	5.0
Giant‐cell tumor of bone	1	5.0
Follicular neoplasm	1	5.0
Malignant Peripheral Nerve Sheath Tumor	1	5.0

### 3.2 Tumor Resection and Chest Wall Reconstruction With Short‐Term Safety Analysis

All patients underwent chest wall wide excision to achieve adequate surgical margins. Specific details of surgical resection and reconstruction were shown in Table 2. After tumor resection, planned chest wall reconstruction was performed to repair chest wall defects. No perioperative or hospitalization‐related deaths occurred within 28 days. Major postoperative complications included surgical site infection (3 cases, 15%), pneumonia (2 cases, 10%), and pleural effusion (1 case, 5%). Additionally, one patient developed myocardial infarction 25 days post‐surgery but received timely treatment at the cardiology department, with a favorable prognosis.

**TABLE 2:** Therapeutic strategies and complications of 20 patients.

	N	%
Median Follow‐up Time	35.3 months	
3DP implant shape		
Horizontal type rib	2	10.0
E type rib	0	0
Vertical type rib	4	20.0
Whole sternum	2	10.0
Inferior segment sternum	6	30.0
Upper segment sternum	6	30.0
Reconstruction		
Mesh +3DP implants	15	75.0
Mesh +3DP implants + musculocutaneous flap	5	25.0
Neoadjuvant therapy		
Yes	2	10.0
No	18	90.0
Adjuvant therapy		
Yes	5	25.0
No	15	75.0
Perioperative complications		
Surgical Site Infection	3	15
Pneumonia	2	10
Implant Displacement	0	0
Pleural Effusion	1	5
Myocardial Infarction	1	5
Long‐term adverse reactions (up to June 2025)		
Abdominal wall hernia	1	5.0
Poor wound healing	3	15.0
Incisional pain	1	5.0
Pleural effusion	1	5.0
Sinus Tract	1	5.0
Implant Displacement	2	10.0
Implant Removal Indicated	1	5.0
None	10	50.0
Recurrence and metastasis status (up to June 2025)		
Lung	2	10.0
Bilateral Axillary Lymph Nodes and Ribs	1	5.0
Chest Wall and Liver	1	5.0
None	16	80.0

### 3.3 Long‐Term Safety, Respiratory Function and QOL Assessment After PEEK Prosthesis Implantation

**FIGURE 3:**
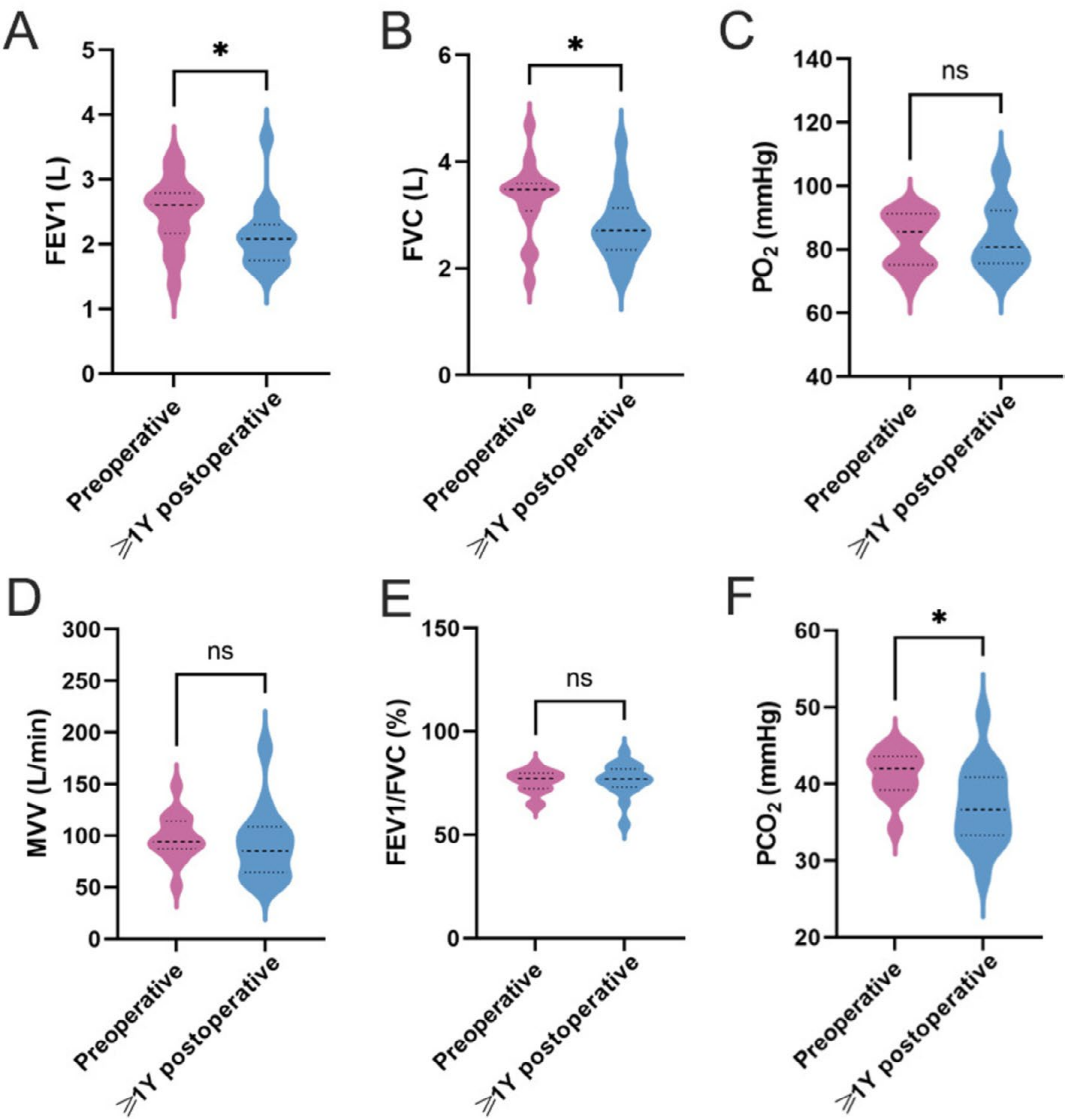
Comparison of pulmonary function and blood gas before and ≥ 1 year after surgery. Pulmonary function indicators (A, B, E, *n* = 16; D *n* = 13): (A) FEV1, (B) FVC, (D) MVV, (E) FEV1/FVC. Blood gas analysis (*n* = 12): (C) PO2, (F) PCO2. *p < 0.05. ns, not significant.

**FIGURE 4:**
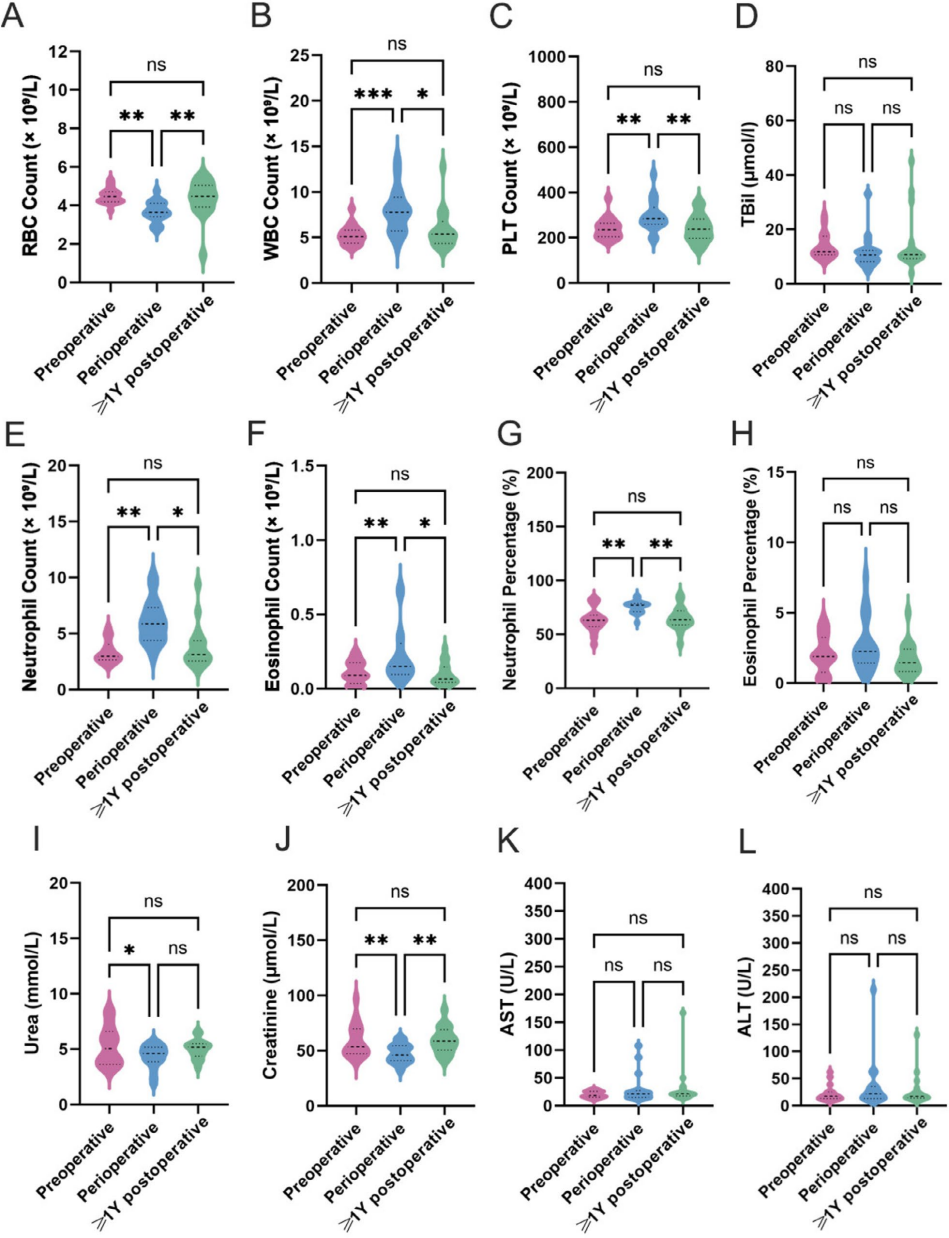
Comparison of preoperative, perioperative (7–28 days postoperative) and long‐term postoperative (≥ 1 year postoperative) blood routine and liver/kidney function tests. Blood routine indicators (*n* = 16): (A) RBC (B) WBC (C) PLT (E) Neutrophil count (F) Eosinophil count (G) Neutrophil percentage (H) Eosinophil percentage. Liver/kidney function indicators (*n* = 16): (D) TBil (I) Urea (J) Creatinine (K) ALT (L) AST. *p < 0.05, **p < 0.01, ***p < 0.001, ns, not significant.

**FIGURE 5:**
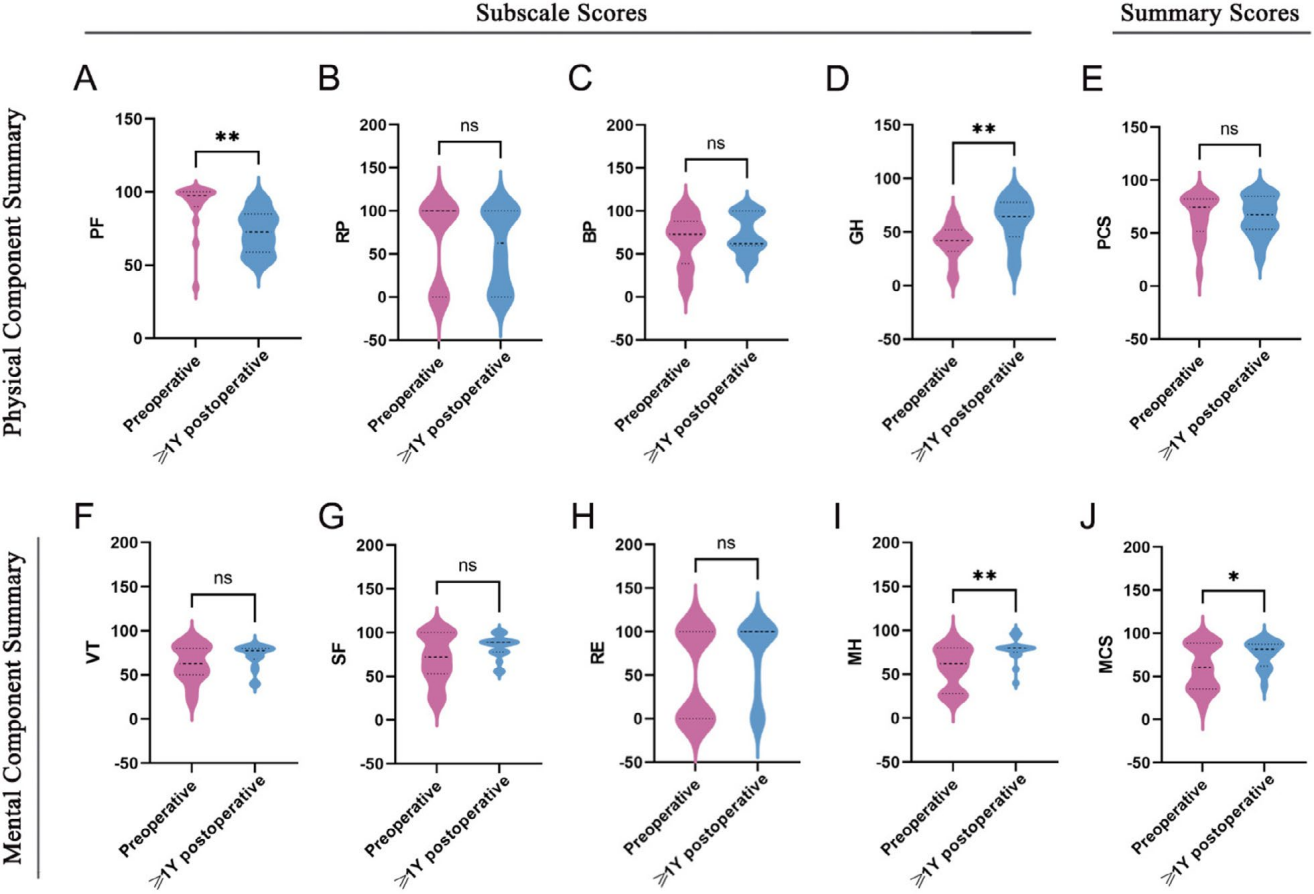
Changes in SF‐36 subscale scores before and ≥ 1 year after surgery (*n* = 18). (A) PF (B) RP (C) BP (D) GH (E) PCS (F) VT (G) SF (H) RE (I) MH (J) MCS. *p < 0.05, **p < 0.01, ns, not significant.

The 20 patients were followed up for a long‐term period, with a median follow‐up duration of 35.3 months (range: 12.7–98.4 months; Table 2). Long‐term safety was evaluated based on long‐term adverse reactions (Table 2) and hepatic and renal function (Figure 4). Respiratory function was assessed using blood gas analysis and pulmonary function tests (Figure 3), and QOL was measured with the SF‐36 scale (Figure 5).

This study collected pulmonary function in 16 patients (MVV in 13 patients) and arterial blood gas parameters in 12 patients. The comparison revealed that 1 year after tumor resection and PEEK prosthesis implantation, FEV1 (Figure 3A) and FVC (Figure 3B) showed significant decreases compared to preoperative values, while MVV (Figure 3D) and FEV1/FVC (Figure 3E) remained stable. Blood gas analysis indicated no significant changes in PO_2_ (Figure 3C) between preoperative and postoperative levels, but a marked difference in PCO_2_ (Figure 3F).

There was a total of 16 paired samples for the results of blood routine and liver and kidney function of the patients. The comparison shows the patient's blood routine test (Red Blood Cell count (RBC, Figure 4A), White Blood Cell count (WBC, Figure 4B), Platelet count (PLT, Figure 4C), Neutrophil count (Figure 4E), Eosinophil count (Figure 4F), percentage of Neutrophils (Figure 4G), percentage of Eosinophils (Figure 4H)) and liver and kidney functions (Alanine Aminotransferase (ALT, Figure 4K), Aspartate Aminotransferase (AST, Figure 4L), Urea (Figure 4I), Creatinine (Figure 4J), Total Bilirubin (TBil, Figure 4D)) changed to a certain extent 7–28 days after the operation, which were transient changes that resolved without specific clinical intervention during the perioperative period. The follow‐up data in ≥ 1 year revealed no significant changes in these parameters as compared with preoperative levels.

Among the 20 patients in this study, one patient developed tumor recurrence and metastasis to the chest wall and liver 6 months post‐surgery and passed away in 2025. Another patient had a prosthesis removed in our hospital in May 2024. During follow‐up in June 2025, it was observed that the prosthesis removal allowed normal mobility, enabling the patient to walk and perform farm work. The remaining 18 patients' QOL was assessed using the SF‐36 scale. Preoperative and 1 year after the operation SF‐36 results are presented in Table 3. Cronbach's α values for all eight dimensions exceeded 0.7, indicating good internal consistency.

**TABLE 3:** Reliability analysis and transformed scores of the SF‐36 scale for 18 patients.

	Preoperative	After 1 year	Dif	*p*
PF	91.1 ± 16.2	72.8 ± 14.0	−18.3 ± 20.3	0.0013
RP	66.7 ± 47.1	56.9 ± 43.1	−4.2 ± 58.5	0.5605
BP	67.0 ± 28.5	72.6 ± 21.9	5.6 ± 29.3	0.4319
GH	40.5 ± 15.9	60.6 ± 20.9	20.0 ± 23.2	0.0019
VT	63.1 ± 20.0	71.1 ± 13.3	8.1 ± 22.4	0.1450
SF	69.8 ± 26.7	83.3 ± 13.0	13.6 ± 27.6	0.0524
RE	50.0 ± 50.0	72.2 ± 41.9	22.2 ± 50.9	0.1406
MH	58.2 ± 22.7	78.7 ± 13.7	20.4 ± 24.4	0.0013
HT	34.7 ± 18.9	63.9 ± 20.8		
PCS	66.3 ± 20.1	65.7 ± 18.1	−0.6 ± 21.8	0.9067
MCS	60.3 ± 25.4	76.3 ± 15.1	16.0 ± 25.8	0.0170

We analyzed the physiological domain of the SF‐36 scale. PF decreased significantly 1 year after the operation compared with that before the operation (Figure 5A), indicating that the patient's ability to perform daily physical activities decreased 1 year after the operation. There was no significant difference in RP, reflecting that the completion degree of work, household chores, etc. remained unchanged due to physical health problems (Figure 5B). A reduction in the BP score (indicating increased pain sensation) was observed, though the difference did not reach statistical significance (Figure 5C). Preoperative pain mainly stemmed from tumor compression, and 65% of patients had tumor‐related pain before surgery (Table 1). After tumor resection, tumor‐induced pain was eliminated, and the slight rise in actual pain level was primarily attributed to surgical trauma. The GH domain showed marked improvement (Figure 5D), indicating enhanced patient perceptions of overall wellbeing and optimistic health prognoses. Weighted scores demonstrated stable PCS levels (Figure 5E), with comparable physiological health status between preoperative baseline and evaluations at ≥ 1 year postoperatively.

We also evaluated the psychological domain of the SF‐36 scale. VT scores demonstrated no statistically significant difference (Figure 5F), indicating comparable energy levels across timepoints. Similarly, SF showed non‐significant variation (Figure 5G), suggesting minimal changes in health‐related social engagement between preoperative and ≥ 1 year postoperative assessments. RE scores remained stable (Figure 5H), reflecting consistent emotional impacts on daily/work activities. However, Table 3 reveals marginal increases in mean scores across these three domains, though the magnitudes were clinically non‐significant. The MH domain (Figure 5I), evaluating anxiety, depression, and emotional stability, exhibited statistically significant improvement postoperatively (*p* < 0.05). This progress translated into elevated MCS scores (Figure 5J), demonstrating enhanced psychological wellbeing at ≥ 1 year follow‐up, likely attributable to alleviation of anxiety/depressive symptoms, increased social participation, and improved emotional regulation capacity.

The HT health change self‐assessment was not included in the statistical results of the SF‐36 scale, which increased from a preoperative mean of 34.7 to 63.9 after 1 year (Table 3), indicating that patients' awareness of their own health status improved.

## 4 Discussion

This study presents long‐term pulmonary function and QOL data, providing strong evidence for the significant role of 3D‐printed PEEK implants in repairing chest wall defects. Such findings are rarely reported in previous literature on chest wall reconstruction. In our study, FEV1 and FVC values decreased significantly in 1 year after surgery, indicating restricted chest wall expansion following surgery and resulting in restrictive ventilatory impairment. No significant difference was noted in MVV, suggesting preserved alveolar ventilation capacity and no marked increase in airway resistance. Arterial blood gas analysis revealed a significant decrease in PCO_2_, indicating enhanced compensatory ventilation postoperatively. This aligns with the early compensatory hyperventilation through increased respiratory rate observed in restrictive ventilatory impairment. PO_2_ showed no significant difference, confirming normal alveolar‐capillary membrane function. No significant differences were noted in blood counts or liver/kidney function between preoperative and one‐year postoperative measurements, confirming the excellent biocompatibility and biosafety of the PEEK implant. In terms of quality of life, the SF‐36 scale was used for assessment. In the physical dimension, PCS remained stable in 1 year after surgery. The surgery led to a limitation in the patient's physical activities, but physical function was not affected. The pain sensation was significantly reduced as compared with preoperative feelings. The patient's overall evaluation of their health improved. This comprehensively demonstrated that the 3D‐printed PEEK implant used for chest wall reconstruction had no negative impact on physical health. In the psychological dimension, the MCS showed a significant improvement in 1 year after surgery, indicating reduced anxiety/depression, increased social participation, and emotional stability.

In a previous study [16], we conducted short‐term pulmonary function follow‐up (from preoperative to 3 months postoperatively) in patients undergoing chest wall reconstruction using 3D‐printed PEEK materials, employing independent samples *t*‐tests for statistical analysis. Results showed a significant decrease in FVC, with the mean value dropping from 2.79 to 2.40 L (difference of 0.39 L), while no significant differences were observed in FEV1/FVC and MVV. In this study, we refined the methodology by employing a paired *t*‐test to compare preoperative and postoperative pulmonary function data from the same patient cohort, while extending follow‐up to after 1 year. Results again demonstrated significant declines in both FVC and FEV1 (mean FVC decrease of 0.30 L), with FEV1/FVC and MVV remaining unchanged. Compared with previous data, the decline trend in FVC was consistent, with a similar magnitude (0.39 L compared to 0.30 L).

Given that pulmonary function measures can be influenced by confounding factors including age and gender, a paired‐sample design was used to account for individual differences and improve the reliability of the results. Extending follow‐up to at least 1 year postoperatively allowed us to better characterize the temporal pattern of pulmonary function changes. In this study, pulmonary function in long‐term follow‐up remained significantly decreased. Notably, in our previous study, we observed a marked decline in pulmonary function at 3 months after surgery. The observed pattern, characterized mainly by reduced FVC with preserved FEV1/FVC, is consistent with restrictive ventilatory dysfunction, which may be attributed to decreased elasticity of the skeletal thorax and surgical injury to respiratory muscles. Such impact on respiratory function is predominantly short‐term. These findings are consistent with the case report [17], in which pulmonary function reached a nadir at 1 month followed by gradual recovery. This consistency supports the notion that the impact of pulmonary function impairment occurs primarily during the early postoperative period, whereas long‐term pulmonary function tends to remain stable.

Previous studies investigating preoperative and postoperative QOL after chest wall reconstruction remain limited [30]. One study evaluated respiratory dynamics and QOL in 23 patients with locally advanced lung cancer or primary/secondary chest wall tumors who underwent conventional non‐rigid reconstruction (NRR) or methyl methacrylate (MMA)‐based rigid reconstruction (RR). Using chest wall motion tracking, the authors reported better respiratory symmetry in the RR group. For quality of life, the EORTC QLQ‐LC13 questionnaire administered at 3–6 months postoperatively showed a slight improvement in pain in the RR group compared with the NRR group, but the difference was not significant, suggesting similar pain relief between the two methods. Another cross‐sectional study [31] demonstrated that patients who underwent traditional chest wall reconstruction had significantly lower pulmonary function and daily activity capacity than healthy individuals. The present study employed a longitudinal, self‐controlled design to evaluate changes in pain and quality of life before and ≥ 1 year after 3D‐printed PEEK implant reconstruction. Compared with prior studies, this research benefits from a longer follow‐up period and a within‐patient comparison, which reduces individual variability and allows a more reliable assessment of the long‐term QOL effects of 3D‐printed PEEK implants.

Our results show that compared with the baseline, there was no significant decrease in the physiological domain score after the operation, while the psychological domain score improved significantly. Personalized 3D‐printed PEEK thoracic and rib reconstruction may improve the mental health of patients. Patients with massive chest wall tumors often present with compromised mental health due to pain and abnormal chest wall morphology. 3D‐printed PEEK reconstruction surgery can relieve these issues. The postoperative improvement in psychological scores seen in our study adds new evidence for the role of 3D‐printed PEEK implants in functional chest wall reconstruction and overall patient recovery. However, due to the limitations of the retrospective design and the small sample size, this exploratory finding still requires validation in further well‐designed clinical studies.

Among 20 patients, one patient in this study underwent prosthesis removal due to prosthesis‐related complications 14 months after surgery. Twelve months after surgery, the patient developed local superficial skin breakdown in the prosthesis implantation area; combined with clinical symptoms and laboratory test results, implant‐related infection was considered. Despite anti‐infective therapy and wound debridement, the chest wall breakdown persisted. To avoid further spread of infection and reduce the risk of long‐term complications, PEEK prosthesis removal was performed. Operation findings determined chronic inflammatory reaction was present around the implant, with hardened fibrous tissue forming a surrounding capsule. No additional rigid reconstruction was performed after implant removal. However, supported by the hardened fibrous tissue, the patient did not develop chest wall paradoxical motion or collapse. The patient has been followed up for a long time to date, with relief of local chest wall symptoms, free movement, and no abnormalities in chest wall function or related discomfort. This prosthesis removal event has clinical enlightenment: in the reconstruction of non‐weight‐bearing bone defects of the chest wall, both PEEK implants and other types of implants may be associated with implant‐related infections. Relevant studies have confirmed that the occurrence of such complications is related to various factors, including local soft tissue conditions, postoperative care quality, and the patient's own immune function status [16, 32, 33, 34, 35]. The patient maintained a certain level of chest wall function and QOL without implanting an alternative prosthesis after PEEK prosthesis removal. It is speculated that the fiber board may play a certain role in chest wall support, replacing part of the prosthesis function, suggesting that the fiber board produced by chronic inflammation may have unrecognized clinical significance, and its mechanism of action in chest wall defect repair is worthy of in‐depth exploration. In the future, we will specifically carry out further research on this issue to provide more evidence‐based medical basis for the management of complications and the optimization of treatment plans in non‐weight‐bearing bone chest wall reconstruction.

The limitations of this study are as follows. First, it was a small‐sample single‐center retrospective study with only 20 cases. Due to the retrospective nature, some patients lacked specific follow‐up data, which were caused by non‐standardized medical record archiving, electronic medical record system updates, and the absence of MVV testing items in local hospitals (Table S1). Although these missing data were non‐selective and did not introduce selection bias, they may still compromise the statistical power of the study. Future research should adopt a multicenter prospective design, standardize the follow‐up testing protocol, expand the sample size, and thus enhance the reliability of the conclusions. Second, the study did not include participants under 18 years old. The average incidence of childhood tumors in China is about 125.72/million [36], and chest wall tumors account for 1.8% of the body's solid tumors [37], of which about two‐thirds are malignant. The most common malignant tumor is Ewing sarcoma, followed by rhabdomyosarcoma and malignant lymphoma [38]. The prognosis for benign tumors in children after resection is relatively good, but the five‐year survival rate for malignant chest wall tumors is only 60%–80% [39]. Children's unique growth and developmental characteristics differ from adults, and the spectrum of tumor diseases may affect the biomechanical compatibility of PEEK implants. Future studies could specifically design cohort studies for adolescents to evaluate long‐term survival outcomes. Thirdly, QOL assessments are subject to confounding factors. Both tumor progression and reconstruction surgery itself may influence patient‐reported outcomes. Future cohorts could exclude tumor‐related QOL interference, focusing exclusively on the long‐term impact of PEEK prosthesis implantation for chest wall defect reconstruction on patients' QOL. Fourth, the absence of a comparator group is another limitation of this study, which limits the ability to specifically attribute the observed functional outcomes to PEEK implants. Due to the single‐center retrospective design of this study, there are certain feasibility limitations in setting a concurrent comparator group, restricted by clinical samples, diagnosis and treatment conditions, and follow‐up resources. Future studies will focus on including comparative cohorts undergoing chest wall reconstruction with alternative materials (such as titanium alloy and polylactic acid), and further clarify the clinical advantages and limitations of PEEK implants through comparative analysis, thereby strengthening the evidence base of the research conclusions.

## Author Contributions

**Ge Li:** conceptualization, methodology, software, data curation, resources, investigation, validation, formal analysis, supervision, funding acquisition, visualization, project administration, writing – original draft, writing – review and editing. **Xiao Liang:** methodology, conceptualization, data curation, software, formal analysis. **Yuanquan Zhang:** formal analysis. **Yangfan Huo:** formal analysis. **Minglei Zhuo:** resources, writing – review and editing, supervision. **Minghai Ma:** formal analysis. **Xing Li:** data curation, formal analysis, methodology. **Lei Wang:** supervision, resources, writing – review and editing, conceptualization, methodology.

## Funding

The authors have nothing to report.

## Disclosure

The authors have nothing to report.

## Conflicts of Interest

The authors declare no conflicts of interest.

## Supporting information


**Table S1:** Detailed List of Missing Data and Specific Causes in 20 Enrolled Patients.

**Data S1:** Supporting Information.

## Data Availability

The data that support the findings of this study are available from the corresponding author upon reasonable request.
