# Supplementary material for: The Long‐Term Quality of Life Study for Patients Received Chest Wall Reconstruction by Using 3D‐Printed PEEK Implants
Source: Thorac Cancer. 2026 Jun 17;17(12):e70323. doi: 10.1111/1759-7714.70323 (PMC13275181; doi:10.1111/1759-7714.70323)
Supplement: Supplementary file 1 — Table S1: Detailed List of Missing Data and Specific Causes in 20 Enrolled Patients. [file TCA-17-e70323-s002.docx]

**Table S1:** **Detailed List of Missing Data and Specific Causes in 20 Enrolled Patients**

| Category of Analysis | Missing number | Specific Causes of Missing Data |
| --- | --- | --- |
| Routine blood | 4 | Non-standardized medical record archiving, data lost  The electronic medical record system updates result in data loss |
| Liver/kidney function | 4 | Non-standardized medical record archiving, data lost  The electronic medical record system updates result in data loss |
| Pulmonary function | 7 (MVV)  4 (Other data) | Follow-up at local hospital; MVV testing item not available  Failed to obtain postoperative ≥1-year pulmonary function results  Non-standardized medical record archiving, data lost |
| Blood gas | 8 | Patient refused blood gas analysis at ≥1-year follow-up  Non-standardized medical record archiving, data lost |
| SF-36 scale | 2 | Surgery in 2021; liver metastasis at 6 months postoperatively; died in 2025  Surgery in March 2023; PEEK implant removal in May 2024 |
